# Supplementary material for: Effects of Age and Popularity of Sport on Differences among Wrestlers’ Parental Support: An Exploratory Study
Source: J Funct Morphol Kinesiol. 2023 May 15;8(2):65. doi: 10.3390/jfmk8020065 (PMC10204366; doi:10.3390/jfmk8020065)
Supplement: Supplementary file 1 [file jfmk-08-00065-s001.zip › supplementary materials-Figures.pdf]

The Effect of Age and The Popularity of Sport on Differences Among Wrestlers in Parental Support

Suplementary files: Figures S1–S4 statistically significant difference in age

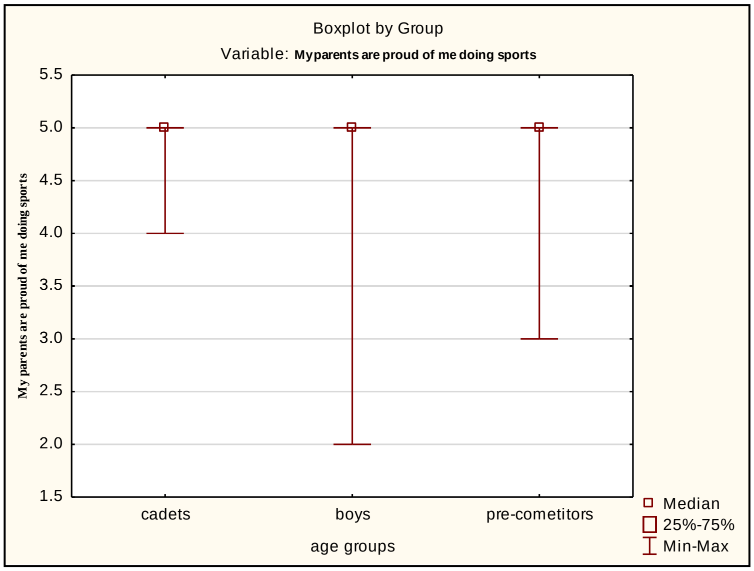

Figure S1 - Item 8 *My parents are proud of me doing sports* [p=0.03]

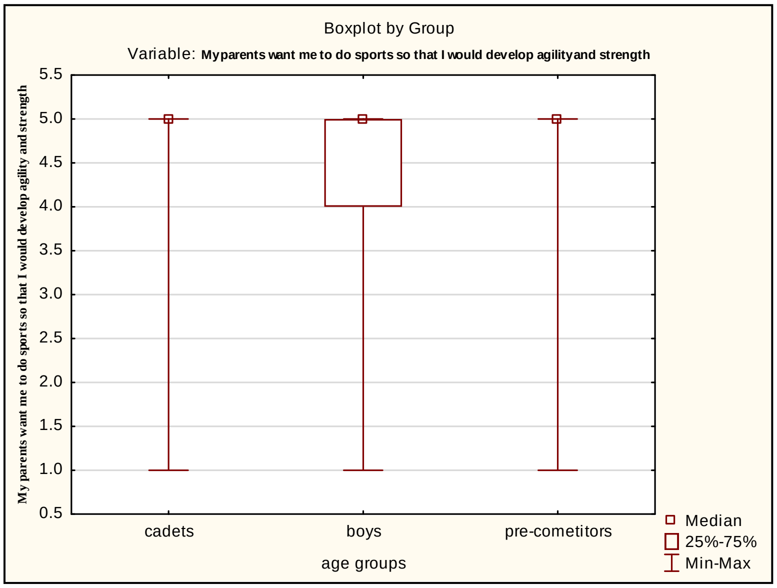

Figure S2 – Item 9 *My parents want me to do sports so that I would develop agility and strength* [p=0.04]

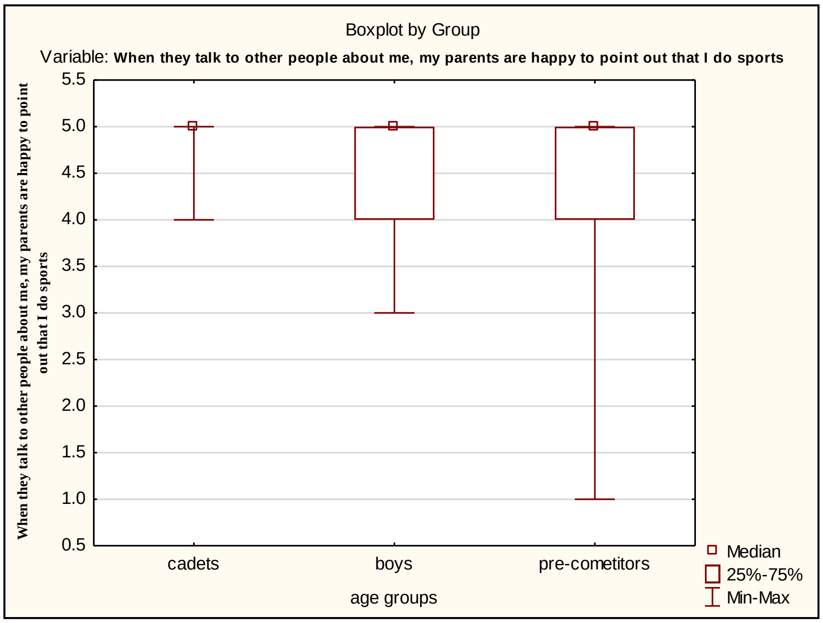

Figure S3 – Item 16 - *When they talk to other people about me, my parents are happy to point out that I do sports* [p=0.03]

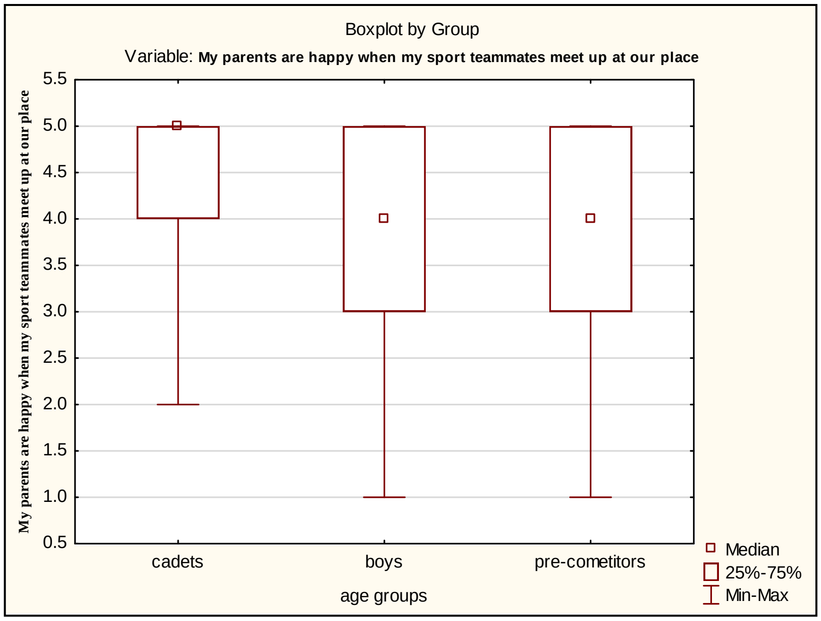

Figure S4 – Item 18 - *My parents are happy when my sport teammates meet up at our place* [p=0.002]

Suplementary files, Figures S5 and S6 statistically significant difference in popularity

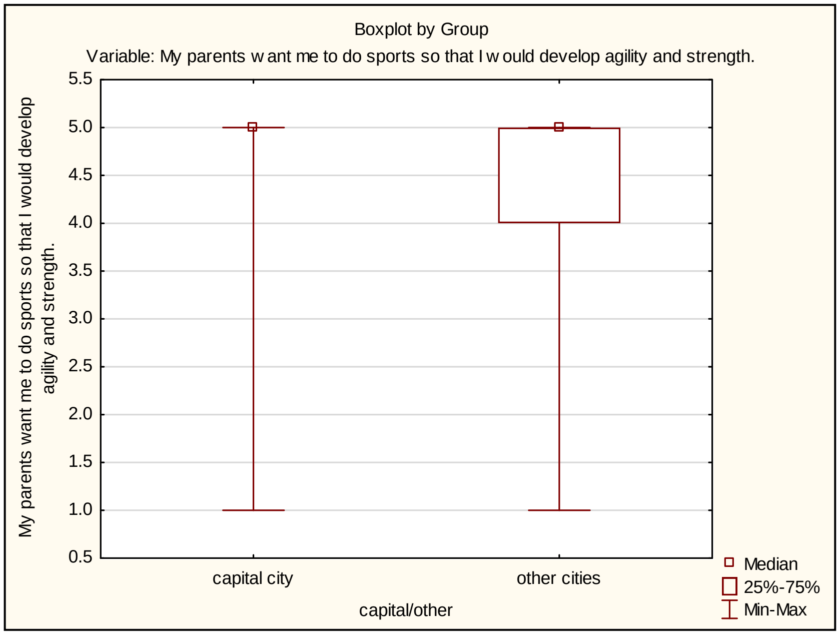

Figure S5 – Item 9 *My parents want me to do sports so that I would develop agility and strength* [p=0.04]

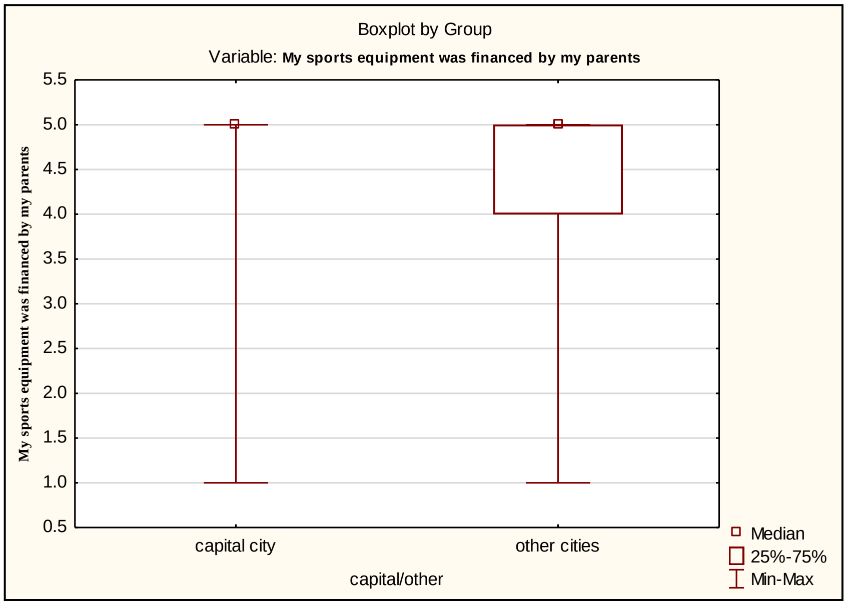

Figure S6 – Item 15 *My sports equipment was financed by my parents* [p=0.03]
